# KIF20A promotes cellular malignant behavior and enhances resistance to chemotherapy in colorectal cancer through regulation of the JAK/STAT3 signaling pathway

**DOI:** 10.18632/aging.102505

**Published:** 2019-12-16

**Authors:** Man Xiong, Kangmin Zhuang, Yunchen Luo, Qiuhua Lai, Xiaobei Luo, Yuxin Fang, Yue Zhang, Aimin Li, Side Liu

**Affiliations:** 1 Guangdong Provincial Key Laboratory of Gastroenterology, Department of Gastroenterology, Nanfang Hospital, Southern Medical University, Guangzhou, Guangdong, China; 2 Department of Gastroenterololgy, Guangdong Second Provincial General Hospital, Guangzhou 510317, China

**Keywords:** colorectal cancer, KIF20A, malignant characteristics, JAK/STAT3, chemotherapy resistance

## Abstract

Background/Aims: Kinesin family member 20A (KIF20A) is upregulated in multiple cancers and plays important roles in promoting malignant behavior, whereas its exact role in CRC remains unknown.

Results: Both genomic and protein expression levels showed that KIF20A was upregulated in CRC. Further functional analyses revealed that KIF20A had a crucial role in improving cell proliferation and resistance to chemotherapy in CRC. Finally, we provided distinct mechanistic evidence that KIF20A achieved all of its pathological functions in CRC by activating the JAK/STAT3 pathway.

Conclusion: Our results suggested that KIF20A regulated a set of malignant characteristics in CRC by activating the JAK/STAT3 pathway. Our findings indicate a new direction for the development of more effective therapeutic treatments for CRC.

Methods: Three Gene Expression Omnibus datasets and The Cancer Genome Atlas datasets were used to investigate the expression level of KIF20A in CRC. Further experiments included immunohistochemical staining, western blot analysis, qRT-PCR, gene silencing, and a cell-injected xenograft mouse model to investigate the interaction between KIF20A and the JAK/STAT3 signaling pathway in both patient-derived specimens and CRC cell lines.

## INTRODUCTION

Colorectal cancer (CRC) is the third most prevalent cancer worldwide and one of the most common tumors of the digestive tract, causing over 50,000 deaths per year [1]. Although progress has been made in the development of therapies, the prognosis of CRC patients remains unpromising, with nearly 50% of patients receiving treatment still dying [2, 3]. CRC is identified as a characteristic malignancy in which genomic aberrations, the tumor microenvironment, and immune responses contribute together for tumorigenesis [4]. A number of studies have reported that dysregulated genes and abnormal signaling pathways participate in the initiation and development of CRC. However, due to the great heterogeneity of CRC, our understanding of the molecular mechanisms underlying CRC is far from ideal. Hence, study of the molecular mechanisms responsible for the initiation and progression of CRC can help to identify potential biomarkers that may facilitate efficient predictive and therapeutic strategies.

Kinesin family member 20A (KIF20A) is a novel member of the kinesin superfamily-6. Kinesin superfamily members have a conserved motor domain that enables them to participate in key cellular processes, such as intracellular transport, mitosis, and migration by interacting with microtubules [5–7]. Previous studies identified that KIF20A is localized to the Golgi apparatus and functions as a motor for the retrograde RAB6-regulated transport of Golgi membranes by interacting with guanosine triphosphate-bound forms of RAB6A/B [8]. Additionally, depending on microtubules and end-directed motility, KIF20A is involved in various cellular processes, such as chromosome partitioning and mitotic spindle formation [5]. Over the last decade, a large body of studies has shown that KIF20A is expressed widely in various organs [9]. In addition, in the field of oncology, the expression level of KIF20A has been found to be upregulated in several types of cancer, including breast, pancreatic, lung, and bladder cancer [10–12]. Furthermore, accumulating evidence has confirmed that KIF20A is capable of regulating malignant behavior in pancreatic and breast cancer [13, 14]. However, our knowledge of the role and underlying mechanism of KIF20A in CRC is limited. In the present study, we discovered the abnormal expression of KIF20A in CRC. With further exploration, we confirmed that the upregulation of KIF20A expression enhanced the malignant characteristics of CRC, including proliferation, metastasis, and chemoresistance. Moreover, we found that KIF20A achieved its pro-tumor function by activating the JAK/signal transducer and activator of transcription 3 (STAT3) signaling pathway.

## RESULTS

### KIF20A is dysregulated in CRC according to online genomic databases

**Figure 1:**
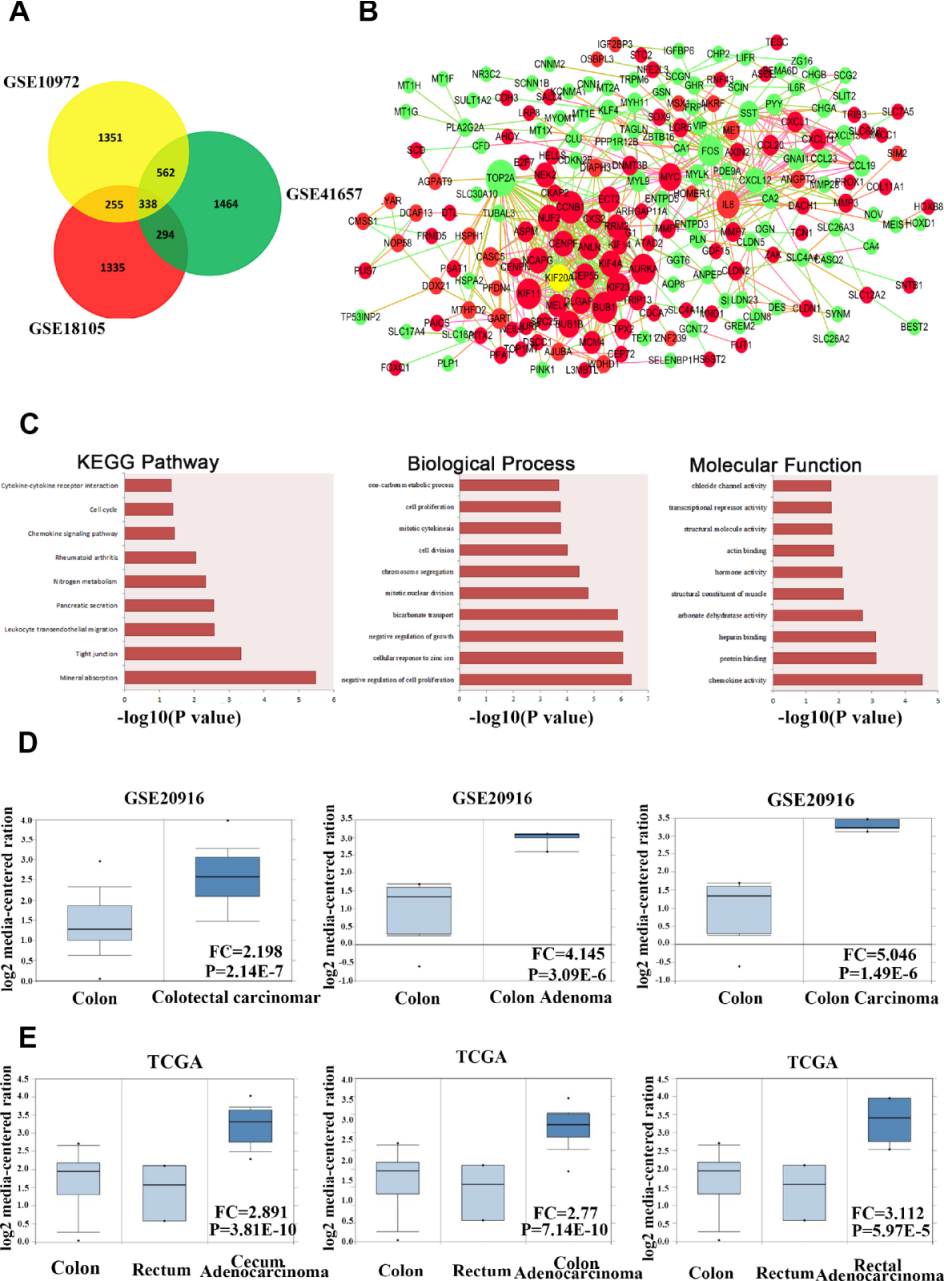
**KIF20A is dysregulated in CRC according to online database information.** (**A**) The overlap of the dysregulated genes from GSE10972, GSE18105 and GSE41657 using GRO2R online analysis. Screen standard: *P* value < 0.05, fold change > 1.5. (**B**) Protein-protein interaction network analysis of 338 overlap genes. Red color represents upregulated genes. Green color represents downregulated genes. Size of the circle reflects the expression fold change of genes. (**C**) Enriched pathway analysis, biological process and molecular function analysis of 338 overlap genes. (**D**) Analysis of the expression pattern of KIF20A in normal colorectal tissue, colon adenoma and CRC based on the data from GSE20916. (**E**) Analysis of the expression pattern of KIF20A in normal colorectal tissue and different types of intestinal cancers based on the data from TCGA.

We used three GENECHIP datasets (GSE10972, GSE18105, and GSE41657) from three sets of clinical specimens to compare gene expression profiles between colorectal carcinoma and normal colorectal tissue, with the help of the GRO2R online tool for filtering and screening differentially expressed genes by setting the Pearson correlation efficient top value as < 0.05 and fold change > 1.5. As a result, 2506 genes from GSE10972, 2658 genes from GSE41657, and 2222 genes from GSE18105 were identified, and the overlap between these three groups generated a total of 338 colon cancer-related genes (Figure 1A).

For further analysis of the gene interaction network, we used the STRING database and found that these 338 genes showed potential physical interactions by forming a complicated multicentric interactive network (Figure 1B). The significantly interacting genes were imported into Cytoscape to calculate the topological features. In the interaction network model, the red or green circles represent upregulated and downregulated genes, respectively. Interestingly, a high connectivity value indicated KIF20A, the yellow circle, as one of the central proteins in the regulatory network with the highest connectivity values.

In order to identify potential signaling pathways or biological processes induced by the 338 overlapping genes, we utilized the KEGG and GO databases for further analysis. In colon cancer, KEGG pathway exploration uncovered significant pathways in mineral absorption, tight junction, leukocyte migration, and pancreatic secretion. GO in biological process and molecular function suggested that the different expressed genes were mostly enriched in several functions, such as regulation of cell proliferation, cellular response to zinc ion, regulation of growth in biological, chemokine activity, and protein and heparin binding, which provided some clues for further mechanistic studies on the role of screened genes in the carcinogenesis and development of colon cancer (Figure 1C).

In addition, we extracted gene expression data for KIF20A from GSE20916 in normal colorectal tissue, benign tumors, and colorectal carcinoma (Figure 1D). The data suggested that KIF20A was expressed at a higher level in both malignant and benign colorectal tumors than in normal tissue. At the same time, KIF20A expression was analyzed in a series of colon cancer cases from the TCGA database between colon, rectum, and different types of tumors (Figure 1E). Surprisingly, it also indicated that KIF20A was expressed at a higher level in CRC than in normal colorectal tissue. Collectively, KIF20A may play key roles in the development and progression of CRC.

### KIF20A is a prognostic predictor in CRC

**Figure 2:**
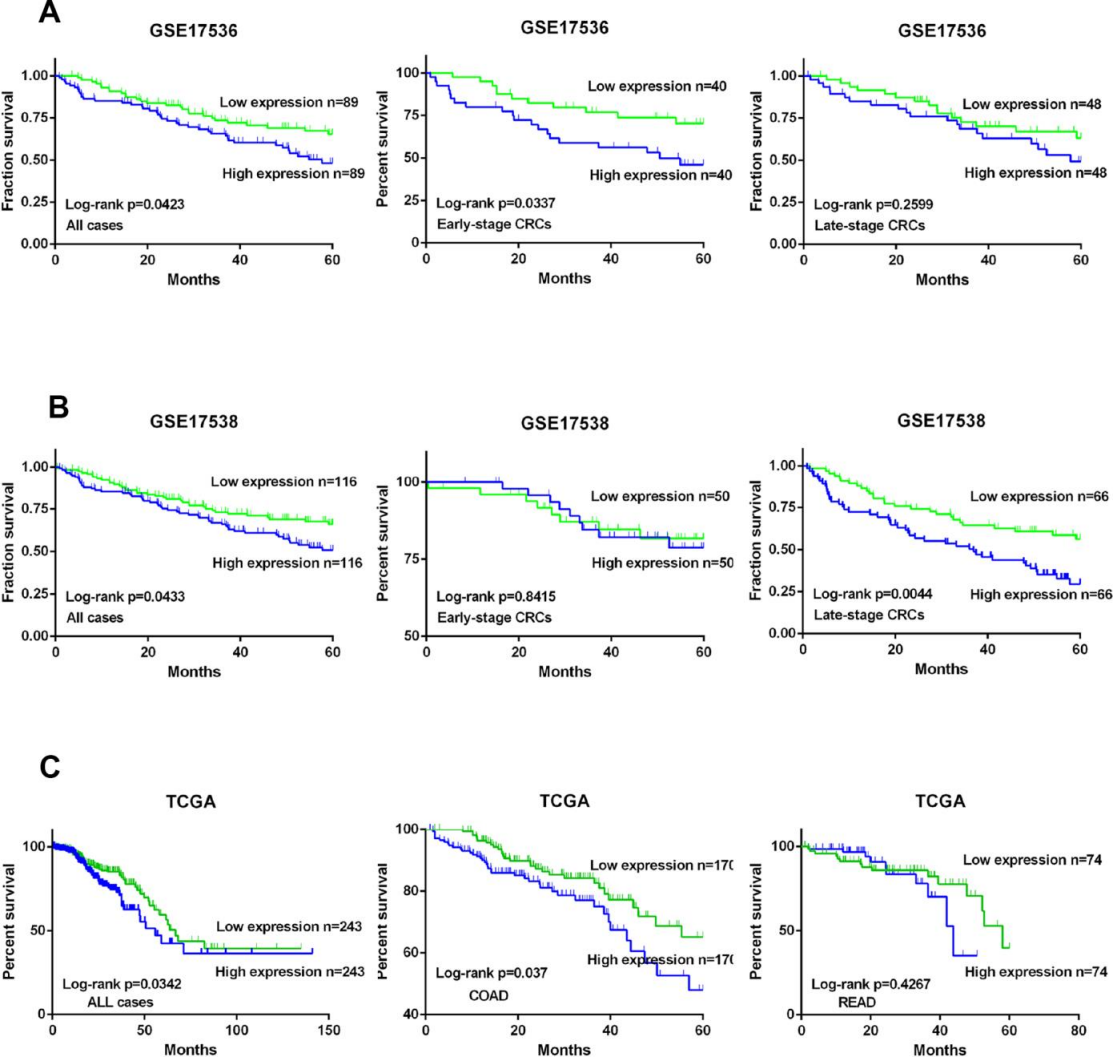
**KIF20A is a prognostic predictor in CRC.** (**A**) Survival analysis of CRC patients with high/low expression of KIF20A based on the data from GSE17536. (**B**) Survival analysis of CRC patients with high/low expression of KIF20A based on the data from GSE17538. (**C**) Survival analysis of CRC patients with high/low expression of KIF20A based on the data from TCGA.

On the basis of data acquired from GSE17536 and GSE17538, we drew Kaplan-Meier (K-M) curves and analyzed the survival of all CRC patients including early- and late-stage CRC. CRC patients were divided into two groups based on the expression level of KIF20A. K-M curves confirmed that the survival time of patients in the high KIF20A expression group was significantly shorter than that of the low KIF20A expression group in GSE17536 (log-rank test, *P* = 0.0423 for overall survival, *P* = 0.016 for early-stage, and *P* = 0.2599 for late-stage, Figure 2A), and GSE17538 (log-rank test, *P* = 0.0433 for overall survival, *P* = 0.8415 for early-stage, and *P* = 0.0044 for late-stage, Figure 2B).

Meanwhile, data acquired from TCGA, including patients with colon adenocarcinoma and patients with rectal adenocarcinoma, were also divided into two groups according to KIF20A expression level (high and low). The K-M curves are shown in Figure 2C. Crucially, survival analysis showed that KIF20A overexpression was significantly associated with poorer survival.

### KIF20A is significantly upregulated in CRC

**Figure 3:**
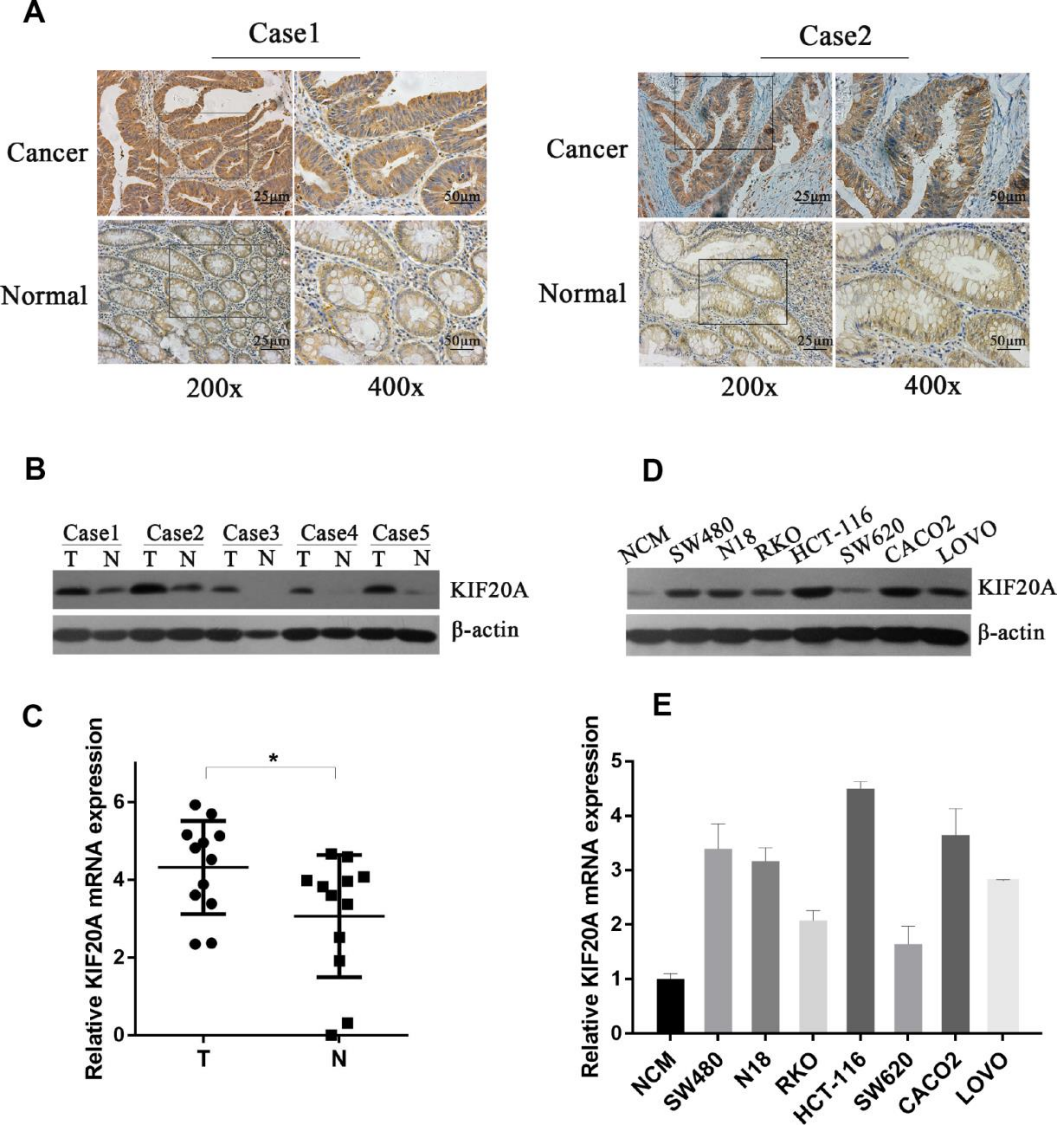
**KIF20A is significantly upregulated in CRC.** (**A**) Immunohistochemical analysis of KIF20A in paraffin tissues from CRC tissues and normal colorectal tissues of two patients (case 1 and case 2). (**B**) Western blot analysis of KIF20A in five pairs of CRC and paracancerous tissues. (**C**) mRNA level of KIF20A in twelve pairs of CRC and paracancerous tissues were detected by RT-PCR. Data are presented as mean ± SEM. *P < 0.05. (**D**–**E**) Western blot and RT-PCR analysis of KIF20A in a normal colorectal cell line (NCM) and seven CRC cell lines. Data are presented as mean ± SEM.

To validate the above findings, we first assessed the expression level of KIF20A in CRC and normal colorectal tissues. KIF20A expression in CRC and paracancerous tissues was analyzed by immunohistochemistry (Figure 3A), and we found that strong staining was observed in the cancer specimens. As for subcellular localization, KIF20A staining was predominantly in the cytoplasm. In paracancerous tissues, the staining was very weak or undetected. To establish further the physiological significance and clinical relevance of KIF20A expression, western blot analysis was perform using fresh-frozen specimens, which confirmed the differential expression of KIF20A in normal colorectal tissues and CRC (Figure 3B and Supplementary Figure 1A). Additionally, KIF20A mRNA expression was assessed in CRC and paracancerous tissues by qRT-PCR (Figure 3C). Taken together, KIF20A expression was significantly upregulated in CRC tissues.

In order to explore further the function of KIF20A *in vitro*, we examined KIF20A expression in seven different colorectal cancer cell lines (SW480, N18, RKO, HCT-116, SW620, CACO2, and LOVO) and one normal cell line (NCM). Consistently, KIF20A expression was higher in the malignant cell lines than in NCM cells (Figure 3D, 3E and Supplementary Figure 1B), which was confirmed by both western blot and qRT-PCR analyses.

### KIF20A improves malignant behavior in CRC

**Figure 4:**
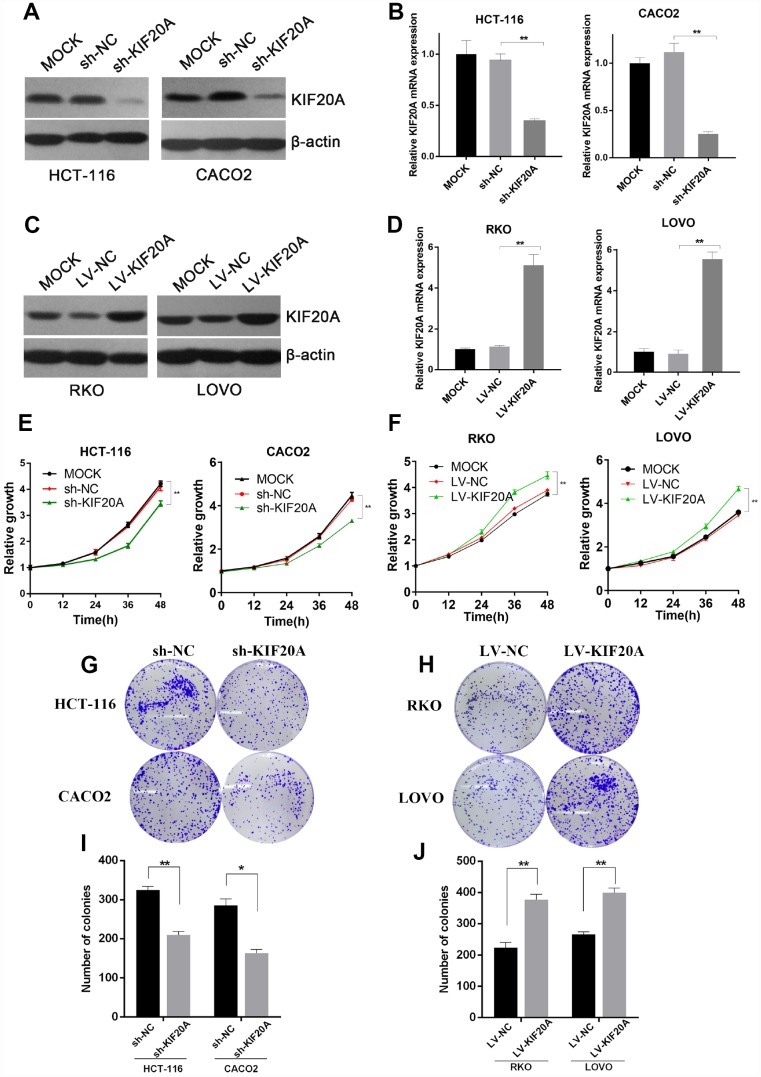
**KIF20A improves malignant behaviors in CRC.** (**A** and **B**) Western blot and RT-qPCR analysis of KIF20A in constructed cell lines. Mock represents blank groups. Sh-NC represents lentivirus-mediated control groups. Sh-KIF20A represents lentivirus-mediated KIF20A silencing groups. (**C** and **D**) Western blot and RT-qPCR analysis of KIF20A in constructed cell lines. Mock represents blank groups. Lv-NC represents lentivirus-mediated control groups. Lv-KIF20A represents lentivirus-mediated KIF20A overexpression groups. (**E**) The relative growth rates in KIF20A knockdown CRCs were measured using CCK-8 analysis and compared between 3 groups at indicated times in two different cell lines. Data are presented as mean ± SEM. *P < 0.05; **P < 0.01. (**F**) The relative growth rates in KIF20A overexpression CRCs were measured using CCK-8 analysis and compared between three groups at indicated times in two different cell lines. Data are presented as mean ± SEM. *P < 0.05; **P < 0.01. (**G**–**J**) Colony formation of 4 different cell lines transfected with different treatments. Data are presented as mean ± SEM. *P < 0.05; **P < 0.01.

As a motor protein, KIF20A has kinetic functions in the cell cycle, cell division, and motility. We chose the two CRC cell lines (HCT-116 and CACO2) with the highest expression level of KIF20A and constructed shRNA-mediated KIF20A-silenced cell lines via lentivirus infection. Knockdown efficiency was confirmed by western blot (Figure 4A and Supplementary Figure 2A) and qRT-PCR (Figure 4B) analyses. CCK-8 and colony formation assays suggested that KIF20A knockdown greatly impaired the proliferative ability of CRC cells (Figure 4E, 4G, and 4I).

Similarly, the RKO and LOVO cell lines, with the lowest expression level of KIF20A, were chosen to construct KIF20A-overexpressing cell lines via lentivirus infection. Lentiviral infection efficacy was confirmed by western blot and qRT-PCR analyses (Figure 4C, 4D and Supplementary Figure 2B). KIF20A overexpression markedly boosted proliferation and clonogenic formation in both of these CRC cell lines (Figure 4F, 4H, and 4J).

**Figure 5:**
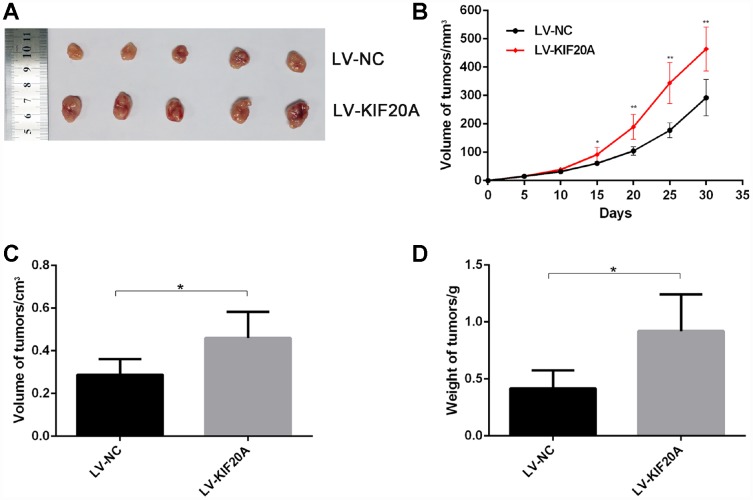
**KIF20A promotes colorectal cancer cells growth in vivo.** The volume and weight of xenografted tumor from different groups were compared. (**A**) Representative images of tumor form in each group. (**B**–**D**) Growth curve of tumors quantification of volume and weight in each group Data are presented as mean ± SEM. *P < 0.05; **P<0.01.

To confirm the effect of KIF20A on CRC carcinogenesis, we performed tumorigenesis assays in nude mice. The tumors formed by KIF20A-transduced CRC cells grew more rapidly and were larger in size than those of the control group (Figure 5A–5D), indicating that KIF20A overexpression also significantly aggravated tumor formation and progression *in vivo*.

### KIF20A induces chemoresistance in CRC

**Figure 6:**
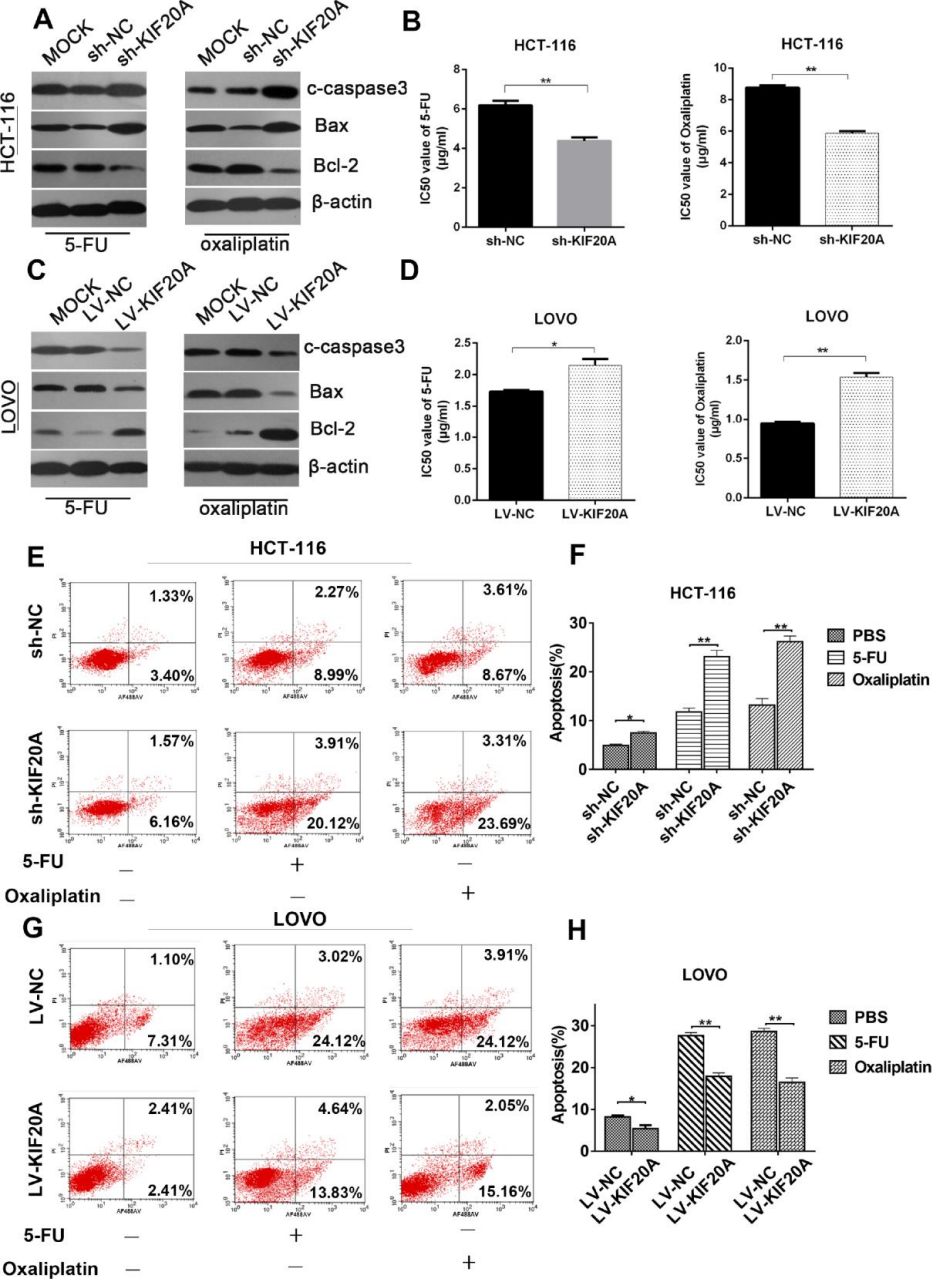
**KIF20A induces chemo-resistance in CRC.** (**A**, **C**) Western blot analysis of apoptosis-related factors (cleaved-caspase 3, bax, bcl-2) in different transfected groups in HCT-116 cell line treated with 4ug/ml 5-FU (left) or 8 ug/ml oxaliplatin (right) and LOVO cell line treated with 1.5 ug/ml 5-FU (left) or 1 ug/ml oxaliplatin (right). (**B**, **D**) Cell viability was measured using CCK-8 analysis and compared between different groups with different treatments at indicated times. (**E**–**H**) The apoptotic rates of different transfected groups with different treatments were measured by flow cytometry. Data are presented as mean ± SEM. *P < 0.05; **P < 0.01.

Furthermore, to explore the possible role of KIF20A in chemotherapy resistance and the underlying mechanisms involved, we investigated the protein levels of apoptosis-related proteins such as cleaved-caspase-3, Bax, and Bcl-2 in KIF20A-overexpressing HCT-116 and LOVO cell lines after treatment with 5-FU and Oxaliplatin. The expression levels of apoptosis-related proteins were obviously downregulated in the KIF20A-overexpressing cell lines, while they were upregulated in the KIF20A-knockdown cell lines (Figure 6A, 6C and Supplementary Figure 3). The IC50 values of 5-FU and Oxaliplatin were greatly improved in the KIF20A-overexpressing cells and impaired in the KIF20A-knockdown cells (Figure 6B and 6D).

For further evaluation, cell growth and apoptosis rate were determined by flow cytometry. The apoptosis rate was upregulated in the KIF20A-knockdown cells, while it was dramatically reduced in the KIF20A-overexpressing cells under treatment with either 5-FU or oxaliplatin, strongly suggesting that KIF20A positively regulates chemoresistance in CRC (Figure 6E–6H). Interestingly, the dysregulation of KIF20A expression affected cell viability even in a control group treated only with PBS (Figure 6F and 6H), further confirming that KIF20A is a critical factor in the survival and development of CRC.

### KIF20A mediates malignant behavior in CRC via the JAK/STAT3 signaling pathway

**Figure 7:**
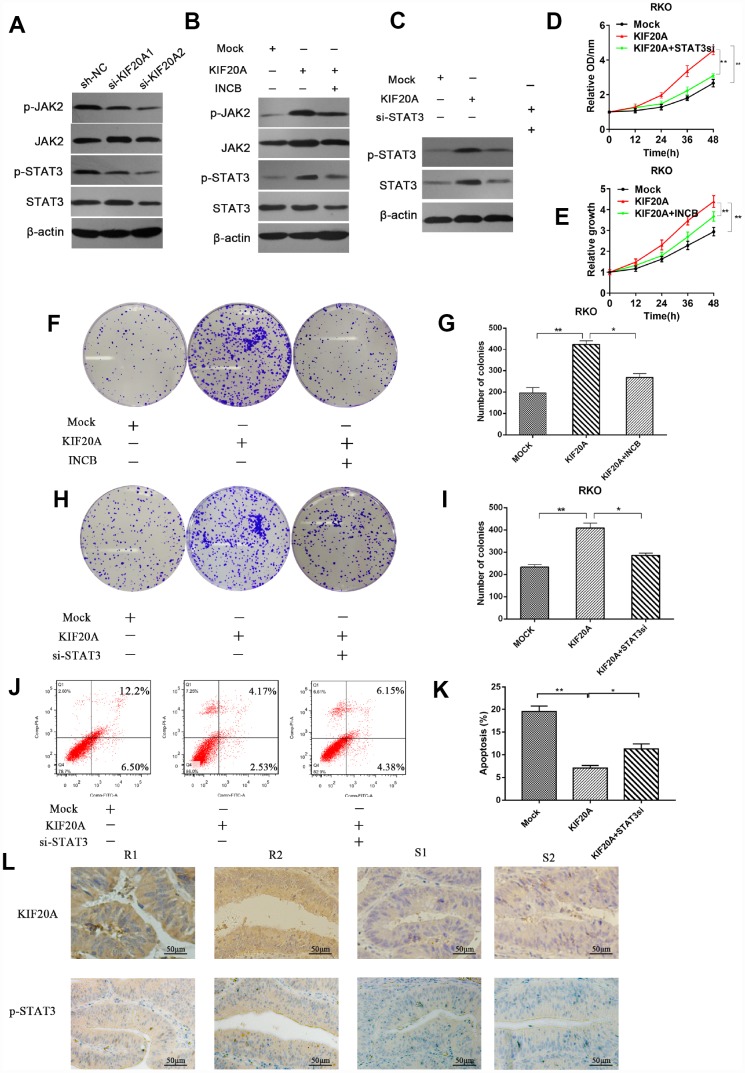
**KIF20A mediates malignant behaviors in CRC via the JAK/STAT3 signaling pathway.** (**A**) Phosphorylation of JAK and STAT3 in different transfected groups was determined by western blot analysis. (**B**) Western blot analysis of phosphorylation of JAK and STAT3 in different transfected groups with or without the administration of JAK inhibitor, INCB. (**C**) Western blot analysis of phosphorylation of JAK and STAT3 in different transfected groups with or without the silence of STAT3. (**D**, **E**) Relative growth rate of different transfected groups with or without the administration of INCB or silence of STAT3. The comparison was made at indicated times. (**F**–**I**) Colon formation assay of different transfected groups with or without the administration of INCB or silence of STAT3. (**J**–**K**) The apoptotic rates of different transfected groups with or without silence of STAT3 were measured by flow cytometry. (**L**) Immunohistochemical staining of KIF20A and p-STAT3 in an independent set of CRC tumor samples with good or poor responses to 5-FU and oxaliplatin therapy. Scale bar=50 μm. Data are presented as mean ± SEM. *P < 0.05; **P < 0.01.

Previous studies reported that STAT3 activation was closely associated with chemoresistance in a number of malignancies including lung cancer and head and neck cancer [15, 16]. Hence, we examined if there was a connection between KIF20A and JAK/STAT3 signaling. We observed that the levels of phosphorylated JAK2 and phosphorylated STAT3 were decreased in KIF20A-knockdown cell lines, while total JAK2 and STAT3 levels remained the same (Figure 7A and Supplementary Figure 4A). We further confirmed that KIF20A overexpression could upregulate the phosphorylation level of JAK2 and STAT3 (Figure 7B and Supplementary Figure 4B). Besides, both the additional administration of the JAK2 inhibitor INCB and knocking down STAT3 could counteract the effect induced by KIF20A overexpression (Figure 7B, 7C and Supplementary Figure 4B–4C).

Subsequently, we explored whether direct interference in the JAK2/STAT3 pathway could affect the function of KIF20A in CRC cell lines. Consistently, we found that the use of INCB or STAT3 knockdown could reverse the increased clonogenicity and proliferative ability induced by KIF20A in the CCK-8 assay and colony formation assay (Figure 7D–7I). In addition, STAT3 silence could abolish the reduced apoptotic rate in KIF20A-overexpressing cells under treatment with 5-FU (Figure 7J, 7K). Furthermore, mFOLFOX6-nonresponsive CRC tissues had high KIF20A and p-STAT3 expression (Figure 7L). Collectively, these findings indicated that KIF20A promotes CRC carcinogenesis by modulating the JAK2/STAT3 signaling pathway.

## DISCUSSION

Colorectal cancer is one of the most lethal malignancies worldwide. Although advances have been made in its early diagnosis and therapeutic strategies, the prognosis of CRC patients is still not promising. KIF20A is involved in various physiological cellular activities, including the intracellular transport of vesicles and organelles and cell mitosis [17]. Although the carcinogenic effect of KIF20A in multiple malignancies has been reported [13, 14], KIF20A has never been studied in relation to the tumorigenesis and progression of CRC. The present study is the first to illustrate clearly the exact role of KIF20A in CRC tumorigenesis.

In our study, we found that KIF20A expression was upregulated in CRC compared to normal colorectal tissue at both the mRNA and protein level, consistent with the data derived from genomic databases. In addition, we investigated the clinicopathologic significance of KIF20A in CRC and found that high KIF20A expression was correlated with poor prognosis in both early- and late-stage CRC patients, indicating that KIF20A may serve as a potential prognostic biomarker in CRC. However, the diagnostic value of KIF20A still needs to be studied further.

Moreover, in order to identify the function of KIF20A in regulating CRC, we performed a series of *in vivo* and *in vitro* studies. Their results demonstrated that KIF20A manipulated the malignant behavior of CRC by enhancing the proliferative potential and clonogenicity of CRC cells.

One of the major problems affecting the prognosis of patients with CRC is resistance to chemotherapy, especially in late-stage patients. Although corresponding therapeutic strategies have been developed in the past few years with the administration of novel FOLFIRI and FOLFOX regimens, the clinical outcome remains unpromising. In our study, we surprisingly discovered that KIF20A could induce chemoresistance in CRC cells and decrease drug-induced apoptosis, which offered a novel explanation for the emergence of chemoresistance in CRC. Thus, KIF20A might be a new therapeutic target to improve the chemoresistance of CRC patients.

The STAT family is a group of important transcription factors that mediate the expression of a series of growth factors and cytokines that take part in various cellular biological activities important for carcinogenesis, including cell survival, cell cycle, and angiogenesis. Among the seven STAT family members, STAT3 is reported most frequently to participate in the regulation of a wide range of cancers including colon, lung, breast, ovarian, and prostate [18–21].

STAT3 is activated by tyrosine kinases such as JAK2. Upon its activation, STAT3 protein dimerizes and translocates into the nucleus and acts as a transcription factor. Noticeably, STAT3 activation has been related to chemoresistance in multiple malignancies [22]. In our study, we found that upregulation of KIF20A expression effectively could activate the JAK2/STAT3 signaling pathway by upregulating the phosphorylation of JAK2 and STAT3. Additionally, the administration of the JAK2 inhibitor INCB or knocking down STAT3 could counteract the increased proliferation and chemotherapy tolerance induced by KIF20A upregulation.

Collectively, our work provides evidence that KIF20A regulates malignant properties and induces chemoresistance in CRC by activating the JAK2/STAT3 signaling pathway, which introduces a novel biomarker for CRC and a therapeutic target for the improvement of chemoresistance in patients with CRC.

## MATERIALS AND METHODS

### Bioinformatics exploration

Human genomic expression datasets were downloaded from the Gene Expression Omnibus database (http://www.ncbi.nlm.nih.gov/geo/) and The Cancer Genome Atlas (TCGA) website (http://www.ebi.ac.uk/microarray-as/ae/). The following bioinformatics analysis procedures were performed: (1) three GENECHIP datasets (GSE10972, GSE18105, and GSE41657) from three sets of clinical specimens were used to compare gene expression profiles between colorectal carcinoma and normal colorectal tissues. With the help of the GRO2R online tool (https://www. ncbi.nlm.nih.gov/geo/geo2r/), differentially expressed genes were chosen with a Pearson correlation efficient top value < 0.05 and fold change > 1.5. The overlap between these 3 groups generated a total of 338 colon cancer-related genes. (2) The STRING online platform was used to examine the potential interactions of the protein products of these genes. The gene regulation networks and protein-protein interactions were visualized during these analyses. (3) All of the genes underwent gene ontology (GO) and Kyoto Encyclopedia of Genes and Genomes (KEGG) analyses on the DAVID platform (https://david.ncifcrf.gov/).

### Cell culture and patient samples

Seven colorectal cancer cell lines (HCT-116, CACO2, RKO, LOVO, SW480, SW620, and N18) and one normal colon cell line (NCM) were purchased from the American Type Culture Collection (Manassas, VA), and cultures were maintained in a humidified 5% CO_2_ atmosphere at 37°C and subcultured when 80% confluence was reached. With approval from the institutional review board of the hospital ethics committee (Nanfang Hospital, Southern Medical University), paraffin-embedded colorectal sections were obtained from the Department of Pathology, Nanfang Hospital.

### Immunohistochemistry and western blot analysis

The cells were fixed with 4% paraformaldehyde for 15 min and then blocked with phosphate-buffered saline (PBS) containing 2% fetal bovine serum, 2% bovine serum albumin, and 0.1% Triton X-100 (Wako Pure Chemicals Industries, Osaka, Japan) for 1 h. Then, the cells were incubated with a primary antibody at 4°C overnight, followed by incubation with a secondary antibody at room temperature for 1 h.

The protein extracts were submitted to western blot analysis using a standard protocol.

The following primary antibodies were used: mouse anti-KIF20A antibody (Santa Cruz Biotechnologies, Dallas, TX), anti-cleaved caspase-3 (Santa Cruz Biotechnologies), anti-Bax (Cell Signaling Techology, Danvers, MA), anti-Bcl-2 (Cell Signaling Technology), anti-phospho-JAK2 (Cell Signaling Technolgoy), anti-JAK2 (Cell Signaling Technology), anti-phospho-STAT3 (Cell Signaling Technology), and anti-STAT3 (Cell Signaling Technology). A mouse anti-β-actin monoclonal antibody (Cell Signaling Technology) was used as a loading control.

### qRT-PCR

The indicated cell lines were cultivated in T75 flasks until 80% confluency, and RNA extraction was carried out using a RNeasy Plus Mini Kit (QIAGEN, Hilden, Germany). One microgram of total RNA was reverse transcribed using a Revert Aid H Minus First Strand cDNA Synthesis Kit (Fermentas International, Inc., SK, Canada), and 5 μL cDNA, corresponding to 10 ng reversed-transcribed RNA, was analyzed using a LightCycler480 with SYBR Green I Master (Roche Diagnostics, Rotkreuz, Switzerland). The primer sequences for *Kif20a* detection were 5-TGTGGGTTTTCCCTGAGTTAGT-3 and 5- GATTTGGGGTCTGTGGTACG-3 (Tm 65°C). For internal normalization, β-actin was amplified with sense: 5-AATCGTGCGTGACATTAAGGAG-3 and antisense: 5-ACGTGTTGGCGTAACAGGTCTT-3. Relative expression was normalized to human β-actin (reference gene) using LightCycler480 software release 1.5, version 1.05.0.39 (Roche Diagnostics).

### RNA interference

For transient mRNA silencing, 7.0 × 10^4^ cells were seeded into 6-well plates and transfected with 2μl KIF20A small interfering RNA (siRNA; Hs_KIF20A_5, sense: 5-GGCCAGGUUUCUGCCAAAATT-3, antisense: 5-UU UUGGCAGAAACCUGGCCTT-3) using the HiPerFect Transfection Reagent (QIAGEN) as described previously, the final concentration of siRNA was 20nM [23]. All Negative Control siRNA (QIAGEN) was used as a negative control. Transfection efficiency was verified by qRT-PCR and immunoblot analysis. After transfection, further functional experiments were made at the optimal time point of RNA silencing.

### Virus production and transfection

The full length KIF20A and the hairpin RNA targeting KIF20A (shKIF20A) were synthesized by RiboBio (Guangzhou, China) and cloned into pReceiver-Lv242 plasmid. The constructed plasmids and lentivirus assembly expression plasmids were co-transfected into 293 T cells. Virus containing supernatants were used to infect CRC cells and selected using 0.5 μg/ml puromycin. qRT-PCR and Western blot assays were performed to validated the transduction efficiency.

### Proliferation, colony formation, and apoptosis assays

Cell Counting Kit-8 (CCK-8) analysis was used to evaluate cell proliferation. The transfected cell lines were cultured in 60-mm dishes with culture medium for 24 h and then with culture medium containing 10% (v/v) CCK-8 for 4 h. Proliferation was determined by absorbance measurement at 450 nm using a microplate reader (Versamax ELISA Microplate Reader; VWR International, Atlanta, GA).

For the colony formation assay, cells at a low density (52.63 cells per cm^2^) were seeded in 6-well plates at normal conditions. The plates were incubated at 37°C in a 5% CO_2_ humidified atmosphere and the medium was replaced every 3–4 days. After 15 days, the colonies were counted.

Apoptosis was measured by using an Annexin V-FITC/Propidium Iodide (PI) Apoptosis Detection Kit (Beyotime, Shanghai, China). After treatment with 5-fluorouracil (5-FU) or oxaliplatin at the indicated concentrations for 24 h, the cells were harvested by trypsinization and washed with PBS. Then, the cells were centrifuged and resuspended in an annexin V binding buffer. After incubation with annexin V-FITC/PI, the cells were analyzed by flow cytometry and visualized with an AxioVision scanning system with FloMax software (Partec GmbH, Münster, Germany).

### Subcutaneous tumor xenograft assay

Female BALB/c nude mice were inoculated subcutaneously in the right flank with 2.0 × 10^6^ cells (n = 5 per experimental group). Tumor growth was observed and measured weekly. Tumors were removed according to the schedule.

### Statistical analysis

Student’s t test and Spearman’s rank correlation coefficient were performed with GraphPad Prism 6 (GraphPad Software, Inc., La Jolla, CA). Statistical significance was established at the *P* < 0.05 level in all analyses. Experiments were conducted in triplicate and three independent experiments were carried out. Data are presented as the mean ± standard error of the mean.

## Supplementary Material

Supplementary Figures

## References

[1] Khong TL, Thairu N, Larsen H, Dawson PM, Kiriakidis S, Paleolog EM. Identification of the angiogenic gene signature induced by EGF and hypoxia in colorectal cancer. BMC Cancer. 2013; 13:518. 10.1186/1471-2407-13-518 24180698 PMC4228238

[2] Guthrie GJ, Roxburgh CS, Horgan PG, McMillan DC. Does interleukin-6 link explain the link between tumour necrosis, local and systemic inflammatory responses and outcome in patients with colorectal cancer? Cancer Treat Rev. 2013; 39:89–96. 10.1016/j.ctrv.2012.07.003 22858249

[3] Siegel R, Naishadham D, Jemal A. Cancer statistics, 2012. CA Cancer J Clin. 2012; 62:10–29. 10.3322/caac.20138 22237781

[4] Aran V, Victorino AP, Thuler LC, Ferreira CG. Colorectal Cancer: Epidemiology, Disease Mechanisms and Interventions to Reduce Onset and Mortality. Clin Colorectal Cancer. 2016; 15:195–203. 10.1016/j.clcc.2016.02.008 26964802

[5] Verhey KJ, Hammond JW. Traffic control: regulation of kinesin motors. Nat Rev Mol Cell Biol. 2009; 10:765–77. 10.1038/nrm2782 19851335

[6] Hirokawa N, Noda Y, Okada Y. Kinesin and dynein superfamily proteins in organelle transport and cell division. Curr Opin Cell Biol. 1998; 10:60–73. 10.1016/S0955-0674(98)80087-2 9484596

[7] Hirokawa N, Noda Y, Tanaka Y, Niwa S. Kinesin superfamily motor proteins and intracellular transport. Nat Rev Mol Cell Biol. 2009; 10:682–96. 10.1038/nrm2774 19773780

[8] Echard A, Jollivet F, Martinez O, Lacapère JJ, Rousselet A, Janoueix-Lerosey I, Goud B. Interaction of a Golgi-associated kinesin-like protein with Rab6. Science. 1998; 279:580–85. 10.1126/science.279.5350.580 9438855

[9] Lai F, Fernald AA, Zhao N, Le Beau MM. cDNA cloning, expression pattern, genomic structure and chromosomal location of RAB6KIFL, a human kinesin-like gene. Gene. 2000; 248:117–25. 10.1016/S0378-1119(00)00135-9 10806357

[10] Imai K, Hirata S, Irie A, Senju S, Ikuta Y, Yokomine K, Harao M, Inoue M, Tomita Y, Tsunoda T, Nakagawa H, Nakamura Y, Baba H, Nishimura Y. Identification of HLA-A2-restricted CTL epitopes of a novel tumour-associated antigen, KIF20A, overexpressed in pancreatic cancer. Br J Cancer. 2011; 104:300–07. 10.1038/sj.bjc.6606052 21179034 PMC3031900

[11] Kikuchi T, Daigo Y, Katagiri T, Tsunoda T, Okada K, Kakiuchi S, Zembutsu H, Furukawa Y, Kawamura M, Kobayashi K, Imai K, Nakamura Y. Expression profiles of non-small cell lung cancers on cDNA microarrays: identification of genes for prediction of lymph-node metastasis and sensitivity to anti-cancer drugs. Oncogene. 2003; 22:2192–205. 10.1038/sj.onc.1206288 12687021

[12] Ho JR, Chapeaublanc E, Kirkwood L, Nicolle R, Benhamou S, Lebret T, Allory Y, Southgate J, Radvanyi F, Goud B. Deregulation of Rab and Rab effector genes in bladder cancer. PLoS One. 2012; 7:e39469. 10.1371/journal.pone.0039469 22724020 PMC3378553

[13] Stangel D, Erkan M, Buchholz M, Gress T, Michalski C, Raulefs S, Friess H, Kleeff J. Kif20a inhibition reduces migration and invasion of pancreatic cancer cells. J Surg Res. 2015; 197:91–100. 10.1016/j.jss.2015.03.070 25953216

[14] Khongkow P, Gomes AR, Gong C, Man EP, Tsang JW, Zhao F, Monteiro LJ, Coombes RC, Medema RH, Khoo US, Lam EW. Paclitaxel targets FOXM1 to regulate KIF20A in mitotic catastrophe and breast cancer paclitaxel resistance. Oncogene. 2016; 35:990–1002. 10.1038/onc.2015.152 25961928 PMC4538879

[15] Kulesza DW, Carré T, Chouaib S, Kaminska B. Silencing of the transcription factor STAT3 sensitizes lung cancer cells to DNA damaging drugs, but not to TNFα- and NK cytotoxicity. Exp Cell Res. 2013; 319:506–16. 10.1016/j.yexcr.2012.11.005 23149124

[16] Bourguignon LY, Earle C, Wong G, Spevak CC, Krueger K. Stem cell marker (Nanog) and Stat-3 signaling promote MicroRNA-21 expression and chemoresistance in hyaluronan/CD44-activated head and neck squamous cell carcinoma cells. Oncogene. 2012; 31:149–60. 10.1038/onc.2011.222 21685938 PMC3179812

[17] Goldstein LS, Philp AV. The road less traveled: emerging principles of kinesin motor utilization. Annu Rev Cell Dev Biol. 1999; 15:141–83. 10.1146/annurev.cellbio.15.1.141 10611960

[18] Burke WM, Jin X, Lin HJ, Huang M, Liu R, Reynolds RK, Lin J. Inhibition of constitutively active Stat3 suppresses growth of human ovarian and breast cancer cells. Oncogene. 2001; 20:7925–34. 10.1038/sj.onc.1204990 11753675

[19] Blaskovich MA, Sun J, Cantor A, Turkson J, Jove R, Sebti SM. Discovery of JSI-124 (cucurbitacin I), a selective Janus kinase/signal transducer and activator of transcription 3 signaling pathway inhibitor with potent antitumor activity against human and murine cancer cells in mice. Cancer Res. 2003; 63:1270–79. 10.1038/sj.onc.1208470 12649187

[20] Mora LB, Buettner R, Seigne J, Diaz J, Ahmad N, Garcia R, Bowman T, Falcone R, Fairclough R, Cantor A, Muro-Cacho C, Livingston S, Karras J, et al. Constitutive activation of Stat3 in human prostate tumors and cell lines: direct inhibition of Stat3 signaling induces apoptosis of prostate cancer cells. Cancer Res. 2002; 62:6659–66. 12438264

[21] Mora LB, Buettner R, Ahmad N, Bassel Y, Jove R, Seigne JD. Prostate adenocarcinoma: cellular and molecular abnormalities. Cancer Control. 2001; 8:551–61. 10.1177/107327480100800612 11807425

[22] Katsha A, Arras J, Soutto M, Belkhiri A, El-Rifai W. AURKA regulates JAK2-STAT3 activity in human gastric and esophageal cancers. Mol Oncol. 2014; 8:1419–28. 10.1016/j.molonc.2014.05.012 24953013 PMC4254172

[23] Kamali K, Korjan ES, Eftekhar E, Malekzadeh K, Soufi FG. The role of miR-146a on NF-κB expression level in human umbilical vein endothelial cells under hyperglycemic condition. Bratisl Lek Listy. 2016; 117:376–80. 10.4149/BLL_2016_074 27546538

