# Supplementary material for: KIF20A promotes cellular malignant behavior and enhances resistance to chemotherapy in colorectal cancer through regulation of the JAK/STAT3 signaling pathway
Source: Aging (Albany NY). 2019 Dec 16;11(24):11905–21. doi: 10.18632/aging.102505 (PMC6949076; doi:10.18632/aging.102505)
Supplement: Supplementary Figures [file aging-11-102505-s001..pdf]

SUPPLEMENTARY FIGURES

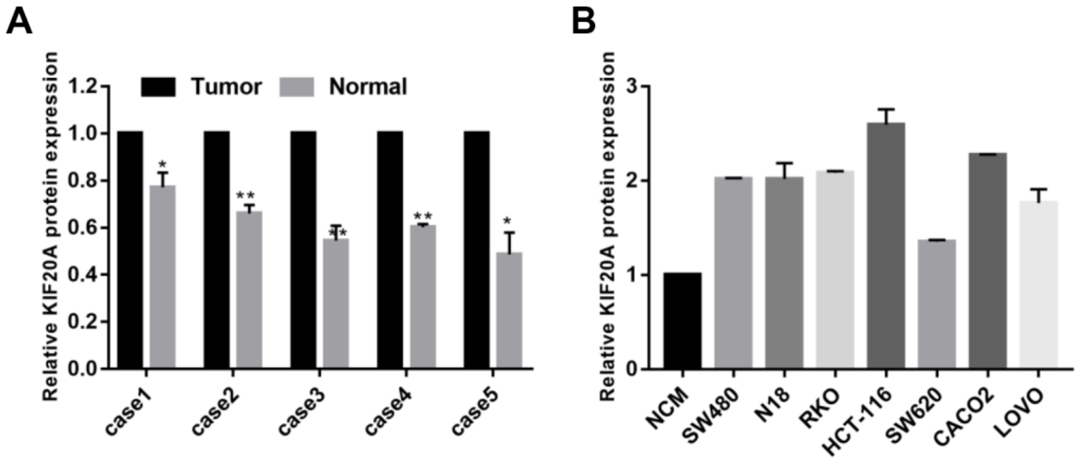

Supplementary Figure 1. (**A**) The protein expression of KIF20A in five pairs of CRC and paracancerous tissues were examed quantitatively. (**B**) The protein expression of KIF20A in n a normal colorectal cell line (NCM) and seven CRC cell lines were examed quantitatively.

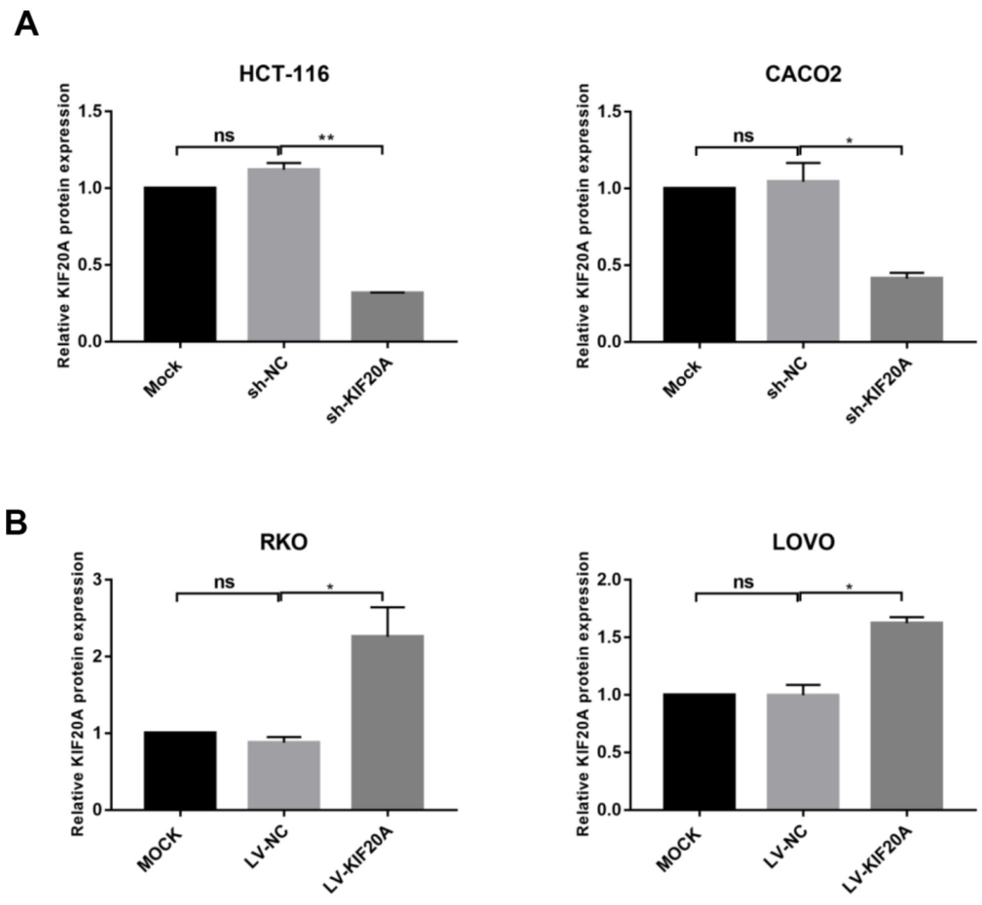

Supplementary Figure 2. (**A**) The protein expression of KIF20A in KIF20A-silenced CRC cells were examed quantitatively. (**B**) The protein expression of KIF20A in KIF20A-overexpression CRC cells were examed quantitatively.

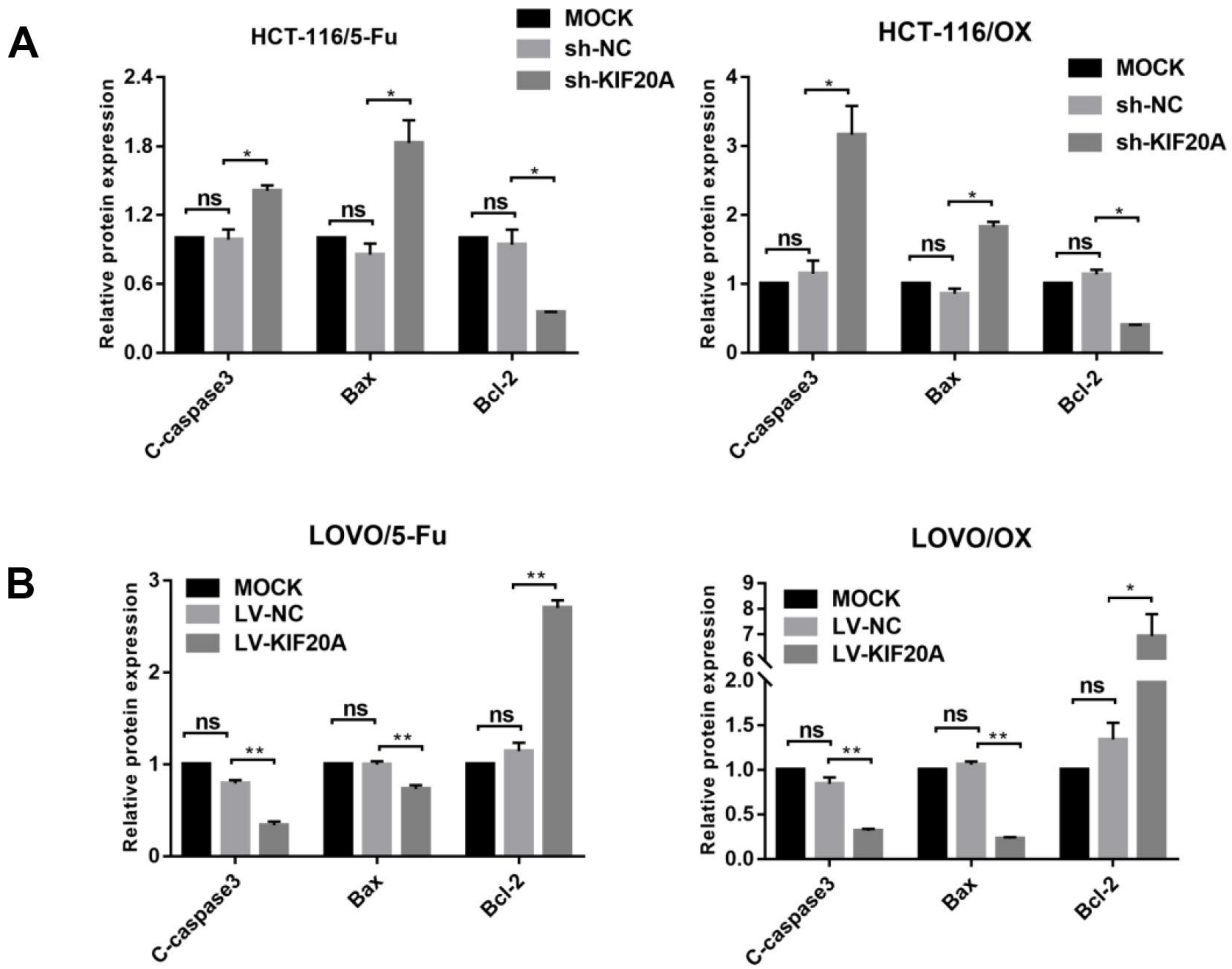

Supplementary Figure 3. (**A**) The protein expression of apoptosis-related factors (cleaved-caspase 3, bax, bcl-2) in different transfected groups of HCT-116 cell line treated with 4ug/ml 5-FU (left) or 8 ug/ml oxaliplatin (right). (**B**) The protein expression of apoptosis-related factors in different transfected groups of LOVO cell line treated with 1.5 ug/ml 5-FU (left) or 1 ug/ml oxaliplatin (right).

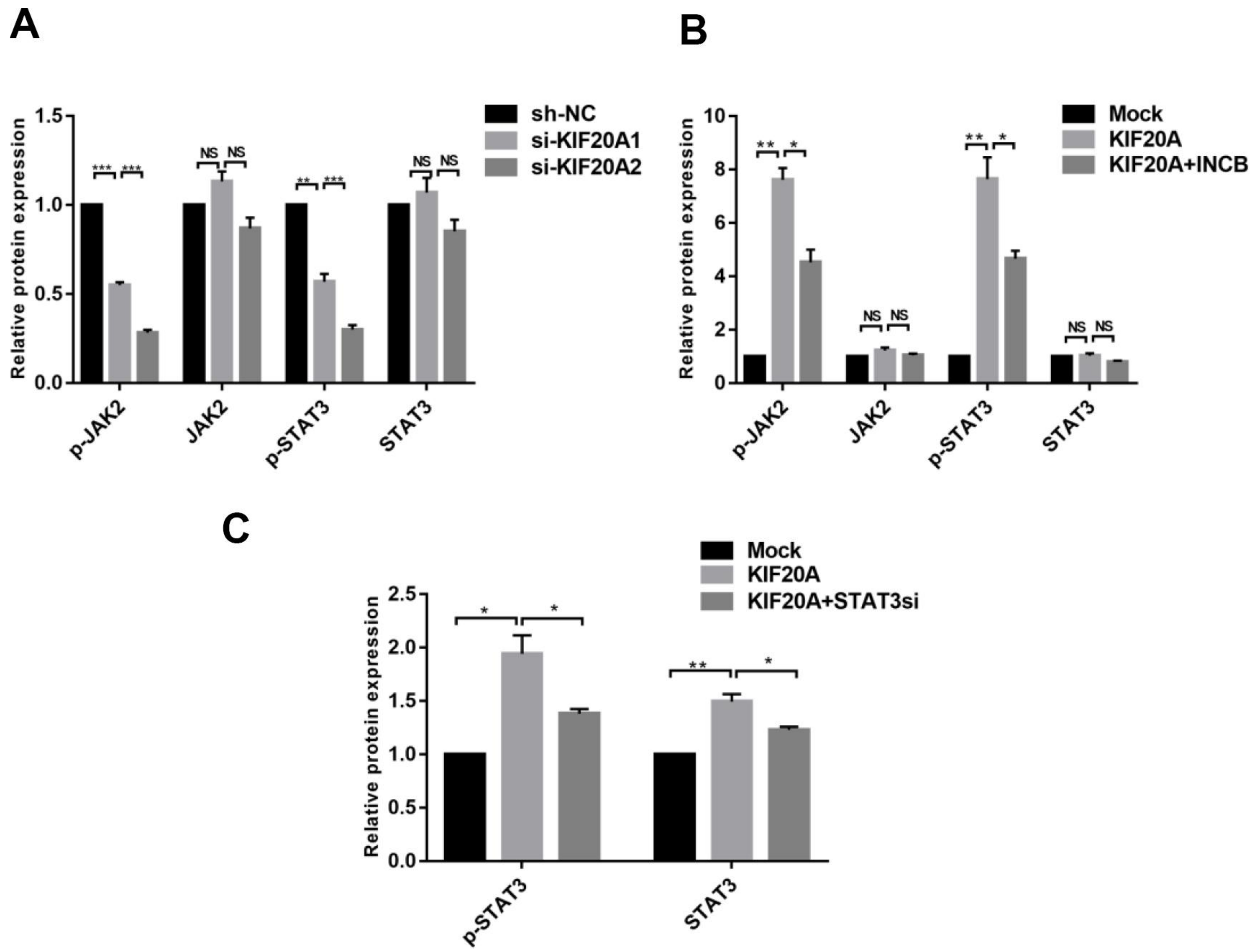

Supplementary Figure 4. (**A**) The protein expression of p-JAK2,JAK,p-STAT3,STAT3 in KIF20A-silenced cells were examed quantitatively. (**B**) The protein expression of p-JAK2,JAK,p-STAT3 and STAT3 in different transfected groups with or without the administration of INCB were examed quantitatively. (**C**) The protein expression of p-STAT3 and STAT3 in different transfected groups with or without the the silence of STAT3.
